# High efficacy of a dimeticone-based pediculicide following a brief application: in vitro assays and randomized controlled investigator-blinded clinical trial

**DOI:** 10.1186/s12895-019-0094-4

**Published:** 2019-10-18

**Authors:** Jorg Heukelbach, Doerte Wolf, John Marshall Clark, Hans Dautel, Kristina Roeschmann

**Affiliations:** 1 Department of Community Health, School of Medicine, Federal University of Ceará, Rua Professor Costa Mendes 1608, 5. andar, Fortaleza, CE 60430-140 Brazil; 2 CardioSec Clinical Research GmbH, Dalbergsweg 21, 99084 Erfurt, Germany; 3 Department of Veterinary & Animal Sciences, Massachusetts Pesticide Analysis Lab, University of Massachusetts Amherst, Amherst, MA 01003 USA; 4 IS Insect Services GmbH, Motzener Straße 6, 12277 Berlin, Germany; 5 G. Pohl-Boskamp GmbH & Co. KG, Kieler Straße 11, 25551 Hohenlockstedt, Germany

**Keywords:** Clinical trial, Head lice, Body lice, Dimeticone, Pediculicidal activity, Ovicidal activity

## Abstract

**Background:**

Increasing resistance of head lice against neurotoxic agents and safety concerns have led to the search for treatment alternatives. Dimeticones with a physical mode of action are safe, and bear a reduced risk for the development of resistance.

**Methods:**

We performed in vitro bioassays to assess pediculicidal and ovicidal activities of a new dimeticone-based product, and a randomized controlled clinical trial to assess efficacy, following 10 min application. Of 153 individuals screened, 100 participants with active head louse infestations were randomly assigned to treatment with either a dimeticone-based test product, or a 0.5% permethrin-based reference product (50 participants per group). Participants received two topical applications of either the test (10 min) or reference products (45 min) at days 0 and 7 or 8. Outcome measures included the efficacies of treatment and their safety, as well as global and local tolerability at baseline, and days 1, 7, and 10.

**Results:**

After 10 min exposure, all lice treated with the dimeticone test product were classified as non-viable in the in vitro assay. Ovicidal activity after treatment of eggs with the dimeticone test product was 96.8%. In the clinical trial, 96 patients completed all study visits. In the full analysis set (FAS) population, on day 1 after one application, 98% of patients were cured in the test group, as compared to 84% cured in the reference group. All participants in both groups were free of head lice on day 10, following two applications (100% cure rate). In total, 42 adverse events (AEs) in 23 patients of both treatment groups were recorded, with the majority of AEs classified as mild.

**Conclusions:**

We have shown a high level of pediculicidal and ovicidal activity, and clinical efficacy and safety, of a brief application of a new dimeticone-based product. The short application time and reduced risk for the development of resistance are key drivers for improved patients’ compliance.

**Trial registration:**

EU Clinical Trials Register EudraCT 2016–004635-20. Registered 14 November 2016.

## Background

Infestation by head lice (*Pediculus humanus capitis*) is one of the most common parasitic diseases in childhood worldwide [1], and there is consensus that infestations have increased during the last decade [2]. This increase has been considered to be caused, in part, by the spread of parasite populations that are no longer susceptible to several pediculicides with neurotoxic modes of action, such as permethrin and malathion, following their extensive use since the middle of the twentieth century [3–8]. Knockdown resistance (kdr) mutations in head lice have been identified to cause insensitivity to pyrethroid treatment [9, 10]. In Germany, kdr-like mutations were found in 93% of head lice investigated, but the majority (92.8%) of children that carried these head lice were treated successfully with permethrin-based products [11, 12].

There is evidence that the occurrence of resistance to neurotoxic pediculicides is increasing. The frequency of kdr-like mutations identified from head lice from all over the U.S. was found to be related to a decrease in the efficacy of a permethrin-based pediculicide (Nix®) [13]. In fact, randomized controlled clinical trials (RCT) conducted in the U.S. three decades ago showed cure rates for pyrethroids of 96–100% [14, 15], whereas more recent RCTs demonstrated cure rates of as low as 25% [16]. Obviously, local and geographical distinct resistance patterns are not yet fully understood.

The need for effective and non-toxic pediculicides has driven investigations for new, effective compounds with good safety profiles, and with a lower risk to induce insecticide resistance. Consequently, physically-acting pediculicides have become more and more popular, with the silicone oil dimeticone as the main active ingredient [17]. Dimeticones are linear polydimethylsiloxanes (CH_3_SiO [SiO(CH_3_)_2_]_n_SI(CH_3_)_2_) of varying chain length. The chain length substantially influences the molecular weight and physical properties of the substance, such as spreading characteristics. Of the various dimeticone-based head louse products available on the market, convincing data on the efficacy and mode of action exist for 4% dimeticone products (Hedrin®/EtoPril®) and a 92% dimeticone product (NYDA®) [18–23].

There is a tendency to shorten the application time in order to increase treatment compliance. Application times as short as 10 min have been described to be effective for products based on mineral oil [24] and neem seed extract [25]. Given the high socio-economic and psycho-social burden of head louse infestations, effective, reliable, safe and rapid treatment options are needed. Contrary to body lice (*Pediculus humanus humanus*), head lice are usually not considered to function as effective vectors for infectious diseases. However, recent studies provided evidence that head lice may serve as vectors for important bacterial pathogens [26].

We performed laboratory and clinical studies to investigate the efficacy and safety of a new rapid-acting dimeticone-based product of the NYDA® family, following a brief application of 10 min.

## Methods

### Test and reference products

The pediculicidal test product (PB790) used for both the in vitro studies and the clinical trial, is a dimeticone-based medical device from the NYDA® product family. All components of the NYDA® formulations have a long history of use in cosmetics and pharmaceutics and are regarded as safe. The dimeticones represent the active component by blocking the respiratory spiracles, thereby inducing death of both lice and eggs by suffocation [23]. The test product was provided by G. Pohl-Boskamp GmbH & Co. KG (Hohenlockstedt, Germany).

A formulation containing 1% permethrin (Nix®, CVS Pharmacy, USA) was used as internal control for the assessment of permethrin resistance of the louse strain in the in vitro bioassay. The active ingredient of the reference product used in the clinical trial is 0.5% permethrin (cis−/trans-relation 25:75) in alcoholic solution (InfectoPedicul®; InfectoPharm Arzneimittel und Consilium GmbH, Germany). Permethrin is a broad-spectrum synthetic pyrethroid and has been widely used for the treatment of head louse infestations in children. InfectoPedicul® and NYDA® are recommended by the Federal Office of Consumer Protection and Food Safety in Germany (Bundesamt für Verbraucherschutz und Lebensmittelsicherheit, 20.10.2015) as disinfestation agents for the treatment of head louse infestation and are thus recommended pursuant to Section 18 of the German Infection Protection Act (IfSG).

### Assessment of pediculicidal activity in vitro

Adult body lice (*Pediculus humanus humanus*) fed on rabbits were kept at 30.0 ± 1.0 °C and 73.8 ± 3.8% relative humidity and used within 24 h after the last feeding. The procedures were applied as described by Oliveira et al. and Sonnberg et al. [27, 28]. In brief, 40–50 ml of the test product were placed into porcelain bowls, and pre-warmed. For every test run, 30 male and female lice were transferred into a plastic sieve. The sieve was placed into the porcelain bowl, and lice were completely immersed in the product for 10 min. Upon immersion, lice were carefully separated from each other using a plastic spatula. As a negative control, identical procedures were followed with demineralized water.

Exposure was stopped by washing the test product off with a 1:4 solution of shampoo (pH Eucerin® Dermo capillaire, Beiersdorf AG, Hamburg) in tap water. The sieve was then dabbed on tissue paper, rinsed with lukewarm tap water for 1 minute and finally again dabbed on a tissue paper. Afterwards, the lice were individually transferred into labelled glass vials containing some gerbil (*Meriones unguiculatus*) hairs as a walking substrate. The vials were left open to observe the lice with a binocular microscope. The time points of observation were: 10, 20, and 30 min, 1, 2, 3, 8 and 24 h after treatment. During monitoring, the viability of each louse was categorized as follows:

- L: Alive and walking
- M: Moribund, reflexes and small movements observed, not walking
- G: Without reflexes, only gut movements are observed
- D: Dead, no movements are observed

Based on published criteria for the assessment of mortality and in compliance to the CRO’s standard protocol, lice in categories M, G and D were defined as non-viable [27, 29, 30]. Tests were repeated three times to obtain a total of 90 lice in each group.

### Assessment of ovicidal activity in vitro

To evidence ovicidal activity of the test product, we performed laboratory tests. Two in vitro hair tuft bioassays were performed. First, lice were immersed in the test and control products, respectively. To simulate more closely the exposure conditions as they exist for in vivo application of the test product, a spray protocol was developed in a second bioassay.

Eggs from permethrin-resistant head lice (*Pediculus humanus capitis*, BR-HL strain), originally collected from infested children in Bristol/UK and maintained on an in vitro rearing system at the University of Massachusetts at Amherst/USA were used in the hair tuft bioassays, similar to the method developed by Strycharz et al. (2012) [31].

For the immersion bioassays, eggs attached to human hair tufts (at least 30 eggs/hair tuft of different developmental stages) were treated with the dimeticone test product or control following an immersing-swirling method for 10 min. A tuft with attached eggs was saturated with the test product by immersing it into 0.5 ml for 30 s with swirling on a small glass slide to ensure saturation and complete egg coverage. As an internal control for permethrin resistance, tufts with attached eggs were saturated for 30 s with 0.5 ml of the 1% permethrin product as above. For a negative control, a dry tuft with attached eggs was saturated with 0.5 ml distilled deionized water (ddH_2_O) as above. After treatments, the tufts were transferred to a clean Petri dish and placed in an incubator (31 °C, 70–80% relative humidity) for 10 min.

Besides immersion, the protocol of the spray bioassays followed the procedures described above, including a 10 min exposure time. Hair tufts with attached eggs were saturated with the dimeticone test product or with ddH_2_O by spraying the formulation until complete egg coverage was achieved by visual observation. The pre-optimized spraying technique using 12 pumps was employed to establish consistent applications. Due to the texture of the internal permethrin control product, the immersion protocol was used for these groups.

At the end of the exposure time, the tufts from both bioassays were shampoo-washed by applying 0.5 ml baby shampoo (Johnson and Johnson, New Brunswick, USA). The shampoo-treated hair tufts were sequentially washed in three separate water baths, each placed on a magnetic stirrer for 40 s per wash and air dried on filter paper for 5 min at room temperature. Dried tufts with treated eggs were placed into covered sterile glass Petri dishes and moved to an incubator at 31 °C, 70–80% relative humidity. Egg viability was recorded daily by examining individual eggs for proper site, shape, and color. The number of lice that hatched from eggs was recorded and used to determine the relative ovicidal activity of the treatments. Underdeveloped eggs and stillborn lice were recorded as dead. All treatments were performed three times.

### Clinical trial

#### Study design/regulatory background

This monocentric, randomized, controlled investigator-blinded trial with a 1:1 allocation ratio was approved by the Ethical Review Board of the Landesärztekammer Thüringen/Germany and by the German Federal Institute for Drugs and Medical Devices (BfArM) by the department for medical devices for the test product, and the department for medicinal products for the reference product, respectively. The design of this study and all assessments performed followed international agreements regarding clinical trials with pediculicides [32]. The primary objective of the RCT was to show that the cure rate, corrected for re-infestation, of the dimeticone treatment is superior to 70% (literature-based lowest acceptance rate). The main secondary objectives were to show that the cure rate of the test product was superior or non-inferior to the reference product, and to assess safety and tolerability of the pediculicidal products tested.

The study protocol followed the ethical principles of the Declaration of Helsinki, ICH-GCP guidelines (International Conference on Harmonization of Technical Requirements for registration of Pharmaceutical for Human Use – Good Clinical Practice), requirements of the German Drug Law including the GCP regulation, and the German Medical Device Law. The trial was registered at the EU Clinical Trials Register (EudraCT2016–004635-20). The study adheres to CONSORT guidelines for reporting clinical trials.

#### Eligibility criteria

Patients of both sexes with active head louse infestations were eligible. Children were included, if they were 2 years of age or older. The reference product was administered according to the most current SPC that was available in Germany before start of the study. For children aged 2 months to 3 years, a maximum dose of 25 ml must not be exceeded, which was strictly followed for children < 3 years. Active infestation was defined as the presence of at least 5 live lice, confirmed by diagnostic combing using combs with a tooth gap of 0.2 mm and tips with blunt parallel-sided teeth.

Patients or their guardians had to be capable of understanding written informed consent form and to give written informed consent after being informed about benefits and potential risks of the trial, as well as details on the insurance covering the subjects participating in the study. Patients had to agree not to use any other anti-lice treatment for the duration of the study. Female patients of childbearing potential had to be tested negative for pregnancy and had to agree to use a reliable method of birth control or remain abstinent during the study.

Patients were excluded if they had used any head lice treatment within the last 30 days prior to the screening visit; used systemic or topical drugs or medications, including systemic antibiotics, which in the opinion of the investigative personnel may interfere with the study results; in the case of allergies or hypersensitivities against any of the active ingredients or the constituents of the products used, skin allergies, multiple drug allergies or multiple allergies to cosmetic products, severe acute scalp disorders, hair longer than mid-back, high probability or known not to follow instructions, or previous participation in this study or in any other investigational trial within the preceding 30 days. Pregnant and breast-feeding women were also excluded.

We did not include patients unable to understand the written and verbal instructions given by the study personnel, in particular regarding the risks and inconveniences. Personnel directly affiliated with this study and/or their immediate families and G. Pohl-Boskamp employees or employees of third-party organizations involved in the study were also excluded.

#### Setting

Recruitment, treatment and diagnostic assessment of patients took place from 18th April, 2017 (first patient in) to 16th March, 2018 (last patient out) at a clinical trial center in Erfurt, Germany, specialized in conducting trials with children and experienced in diagnosis and treatment of head louse infestations. Patients/guardians/caretakers were informed about this study via advertisements, flyers, letters to primary schools and preschools, and internet on-screen displays. All texts used for recruitment had been approved by the responsible ethics committee. Patients/ guardians/caretakers had to contact the study center to arrange an appointment, and after initial assessment of infestation and informed consent procedure, patients were included and allocated to treatment groups.

#### Intervention

Two topical applications, as recommended for treatment of head louse infestations, were performed [32, 33]. The first treatment was applied on day 0 (V1), the second on day 7 or 8 (V3).

Treatments required individual amounts of the investigational products to completely cover hair and scalp of the patient. Both products were applied as recommended by the manufacturers. The test product was evenly sprayed until the hair was completely wetted with the solution and then massaged into the dry (not washed) hair over its full length, with special diligence on the base of the hair near the scalp and the ear region. Curly, long and thick hair was treated in strands. After 10 min without covering, the hair was combed carefully with the nit-comb to remove suffocated lice and eggs. Afterwards the product was washed out with a commercially available shampoo and the hair was rinsed thoroughly.

For application of the reference product, the hair was washed with a commercially-available shampoo and towel-dried. Afterwards, the product was evenly dispensed onto the hair over its full length, again, with special diligence on the base of the hair near the scalp and the ear region. Curly, long and thick hair was treated in strands. After 45 min without covering the hair, the product was washed out with warm water without shampoo. Afterwards, the hair was again carefully towel-dried. Before the hair was completely dry, combing with a nit-comb was performed according to the manufacturer’s instruction for use, to eliminate the eggs. The participants dried their hair by themselves or with the help from their guardians, using a hair dryer.

Metal combs were used, with a gap of 0.2 mm and tips with blunt parallel-sided teeth. The same comb types were used for both groups.

According to the instructions for the reference product, participants were instructed not to wash their hair with shampoo within the next 3 days, to possibly increase the ovicidal efficacy of permethrin. To keep both treatments as similar as possible, the instruction of not washing the hair within 3 days after application was also given for the test group.

#### Informed consent, outcome measures and assessments

The primary outcome was defined as the cure rate at the end of day 10 (V4), corrected for re-infestation. Assessments for head louse infestations were performed on days 1 (V2), 7 or 8 (V3) and 10 (V4), by blinded study staff. Definition of re-infestation was based on current literature: no adult lice or third stage nymphs following first treatment at day 1 (V2), and no more than two adult lice or third stage nymphs found by combing following second treatment on day 10 (V4) [34].

Secondary outcome measures included: global tolerability and local tolerability, rated by the patients and the blinded investigator via 4-point VRS (Tables 4 and 5); skin irritation and eye irritation, assessed by the blinded investigator using a 4-point VRS. The esthetical effect of the products was evaluated (look of hair; feeling of hair; sensation on scalp; categories: “strongly agree”, “agree”, “disagree”, “strongly disagree”) by structured questionnaires. Date, number and type of adverse events were documented.

A total of four assessments were performed (V1-V4). At day 0, during the screening visit, patients were registered and enrolled. After informed consent procedure and signing of the informed consent form (ICF), confirmation of the diagnosis, and assessment for exclusion and inclusion criteria, participants were randomized to one of the two intervention groups. Baseline data (gender, age, hair length, hair type, medical history, concomitant medication) were assessed before treatment. Within 1 hour before first application and 1 hour after application of the respective product, the following assessments were done: assessment of skin irritation, assessment of eye irritation and assessment of adverse events (AE). Additionally, 1 hour after application on V1 and V3 and also during V2 and V4, assessment of global tolerability and local tolerability were performed. These assessments were repeated on days 7 (V3, before and after treatment) and on 10 (V4, final assessments). Application of the questionnaire on esthetical effects was done only after treatment on day 0 (V1) and day 7 (V3).

At all visits after treatment (V2-V4), AEs were recorded for start and end dates and times, seriousness, expectedness, severity, causal relationship to investigational product, and causal relationship to study procedure and evaluated for events per subject and study group. All deteriorations were documented as AEs.

Upon recommendations of the Robert Koch Institute [33] and in line with current state-of-the-art clinical trials [24], patients were allowed to use a nit-comb between study visits. At every visit, patients or their guardians (in the case of minors) were asked if they used the nit-comb provided and answers were documented in the electronic case report form (eCRF).

All assessments of primary and secondary outcome measures were performed by investigators blinded to the treatment applied.

#### Sample size

According to previous preclinical studies with the dimeticone test product and to preclinical and clinical studies with products from the NYDA® family, a 90% cure rate was expected for the test product. A pre-defined limit of 70% was determined from reported cure rates for permethrin-containing products (range from 34.8 to 98.0%, mean 66.6%, approximated to a 70.0% cure rate) and was defined to be the minimal acceptable cure rate [35–38]. A sample size of 42 was required for a one group χ^2^-test comparing cure rate of 90% with a fixed limit of 70% (two-sided test; alpha-level of 0.05; power = 90%; software nQuery advisor 7.0; power = 80%: sample size = 34). Assuming a 10% drop out rate and 5% re-infestation rate, 49 cases would be required in each group, which was rounded up to 50.

#### Randomization

Participants were randomized to one of the two head louse treatments by a computer-generated code using randomly mixed blocks of 10, with a final and random 1:1 allocation. Randomization, enrollment of participants, and assignment of specific participants to one of the two interventions were performed by an investigator not involved with assessment of outcome measures.

#### Blinding

The study was observer-blinded. As both products differ substantially from each other in terms of packaging, smell, application method and exposure time, double-blinding was not possible.

All staff members involved in assessments of primary and secondary outcome measures (investigators and study staff performing the assessments of hair and scalp, eyes, as well the efficacy and safety evaluations) were blinded to treatment assignment. The assessors were not involved in handling, storage, and use of the products, and did not have access to the eCRF entries regarding application of investigational products, such as used amount or start and stop time of exposure.

### Statistical analyses

#### Pediculicidal activity in vitro

At each point of time of observation, the relative frequencies of non-viable lice (categories M + G + D) were calculated for each test run, and the arithmetic mean ± standard deviation (SD)was calculated. Relative frequencies of lice treated with the test product and the water control were compared using Fisher’s exact test using the Statistica® v.7.1 software (StatSoft, Tulsa, USA). For determination of pediculicidal activity, endpoint mortality of lice (mortality determined 24 h after treatment) was compared between test and control.

#### Ovicidal activity in vitro

The mean percent ovicidal activity (± SD) was determined and statistically analyzed using Shapiro-Wilk Test for determination of normal distribution, 2-factorial ANOVA and test for homogeneity of variance (Levenes). Number of replicates was 3 (immersion protocol) and 4 (spray protocol), respectively.

#### Clinical trial

Statistical analysis of data obtained from the RCT followed the predefined statistical and analytical plan (SAP). Descriptive statistics (continuous variables: mean ± SD; ordinal variables: median, min, max, Q1, Q3; categorical variables: counts and relative frequencies) was performed by visit and treatment.

For the primary objective, the following null hypothesis was tested: H_0_prim: pT = 70%. If pT > 70% and if the null hypothesis was rejected by a two-sided, one sample χ2 test at 0.05 level, superiority would be concluded. The primary objective was analyzed in the full analysis set (FAS). The secondary objectives were analyzed in FAS and per-protocol population (PP).

Due to no observed differences for the cure rates after correction for re-infestation (100% efficacy for both treatments), non-inferiority testing was performed in the PP population, with a predefined non-inferiority margin of 7.5%. The following null-hypothesis was tested at an α-level of 0.025. H_0_,NI: pT-pR < δ, whereby δ = − 7.5%. The lower, one sided 97.5% confidence interval of difference pT-pR was used for the test. With a confidence limit of ≥ − 7.5, hypothesis H_0_,NI was rejected and non-inferiority was concluded. The differences of cure rates pT-pR as well the cure rates pT and pR are presented with two-sided 95% confidence interval. NCSS 12.0.2 software was used to calculate the 95% confidence interval for the difference of zero (Miettinen-Nurminen Score).

Cure rates (pT and pR) and difference between cure rates (pDiff = pT - pR) with their two-sided 95% CI including results of two-sided, two sample Fisher’s exact test were calculated. Effect of hair characteristics on efficacy of the products was evaluated via Kruskal-Wallis-Test.

The use of a nit-comb during clinical trials is controversial, as combing may increase efficacy [32]. To estimate the effect of combing, in a post hoc analysis, the association between the occurrence of treatment failures and patients’ implementation of combing was evaluated. For this purpose, treatment failures and combing were evaluated for independence of categorical variables by chi-squared test and by Fisher’s exact test (for small expected frequencies).

## Results

### Pediculicidal activity in vitro

**Fig. 1:**
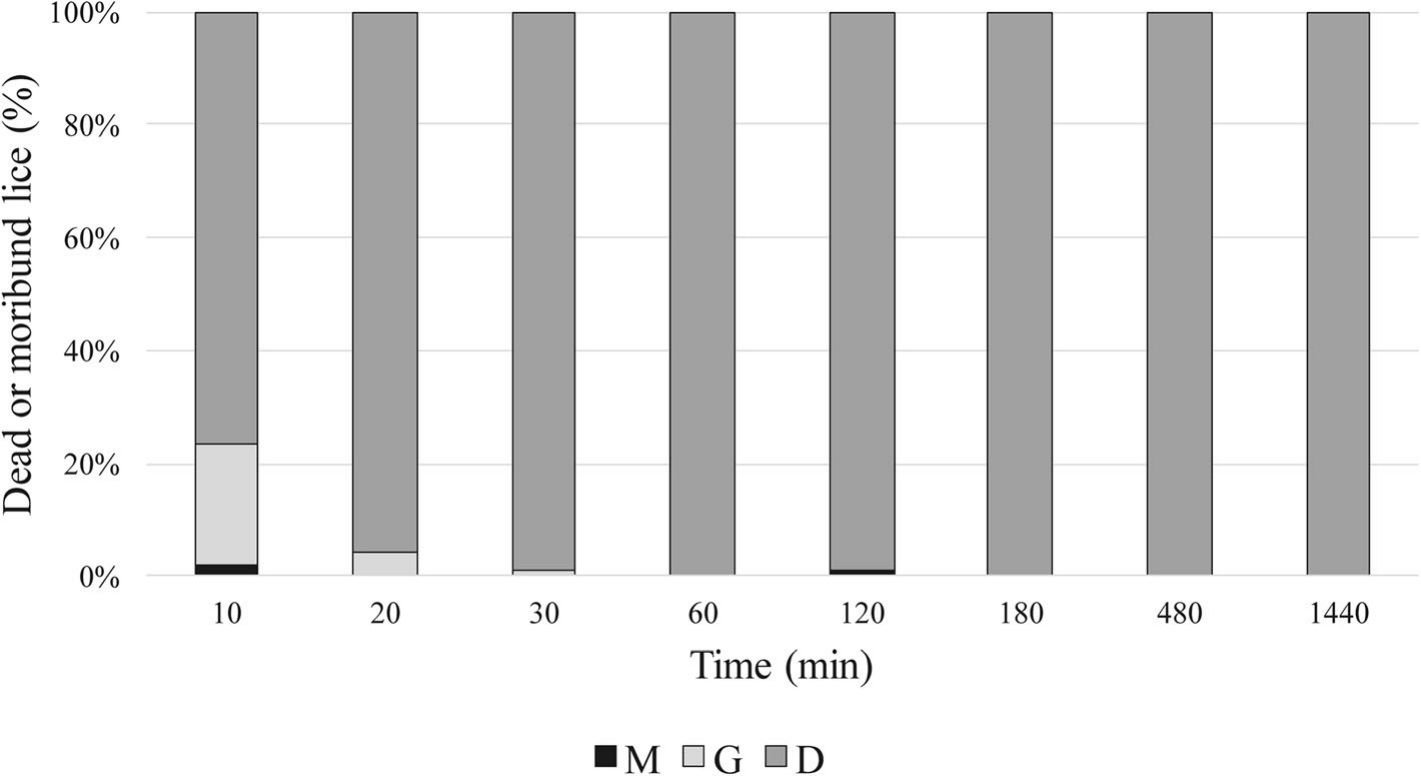
Pediculicidal activity of the test product during monitoring from 10 min to 24 h after treatment, 90 adult body lice per group; Lice fulfilling the categories “M” (moribund), “G” (only gut movements) and “D” (dead) were classified as non-viable

Figure 1 shows the pediculicidal activity of the test product. Ten minutes after treatment, all lice were classified as non-viable (categories M, G or D). After 10 min, only two lice (2.2%) and after 120 min one louse (1.1%) were classified in the category M (moribund, reflexes and small movements observed); at all other observation points, all lice were classified as G or D (i.e. no or only gut movements). No lice recovered from the non-viability status during the observation period of 24 h (Fig. 1).

All control lice were viable 3 hours after treatment. After 8 hours, mortality was only 1.1%. After 24 h, mortality was 7.8% (SD ± 2.4%). The difference in endpoint mortality between treated and control lice was highly significant at all observation points (*p* < 0.001).

### Ovicidal activity in vitro

A total of 98 eggs were tested for ovicidal activity of the test product according to the immersion protocol, and 159 eggs according to the spraying protocol. The use of different treatment protocols did not result in significant differences: mean ovicidal activity after treatment of eggs with the test product was 93.0% (SD ± 4.2%) after immersion, and 96.8% (SD ± 3.7%) after spraying.

Ovicidal activity for the internal permethrin control was 63.1% (SD ± 25.2%) and 56.2% (± 33.2%), respectively. Incubation of the eggs with ddH_2_O as negative control did not result in any considerable ovicidal effect (6.8%; SD ± 3.7% after immersion and 4.2%; SD ± 8.3% after spraying).

### Clinical trial

#### Participants and baseline demographic data

**Fig. 2:**
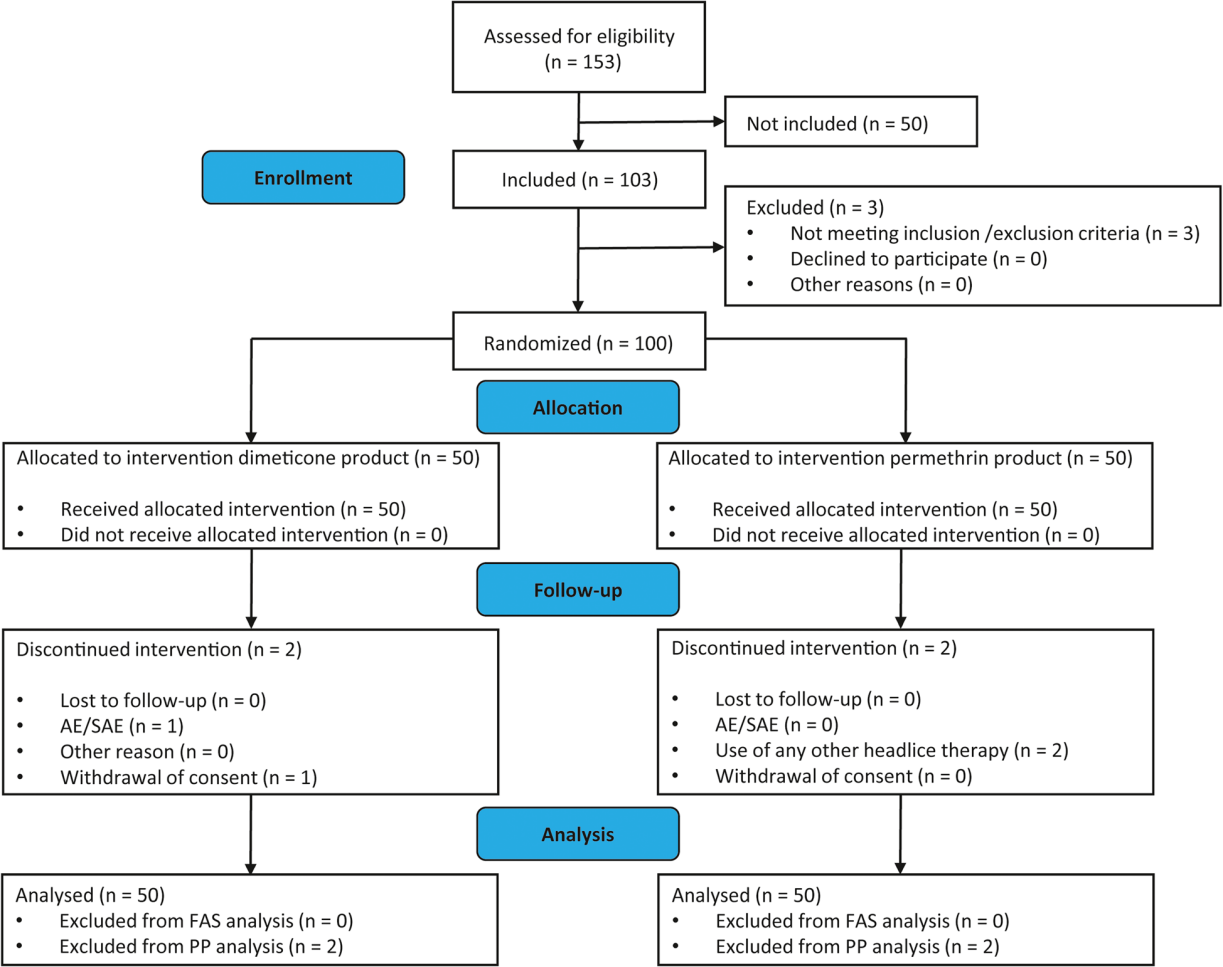
Flow of participants through the trial

The flow of participants through the trial is presented in Fig. 2. Out of 153 subjects assessed, 103 were eligible and screened for head louse infestations. Of these, 100 were enrolled and randomized into one of the two treatment groups. One patient of the test group withdrew consent after day 1 (V2) for unknown reasons and was excluded from the trial.

In total, 96 patients completed all study visits. One patient of the test group dropped out after a serious adverse event (SAE). Due to hospitalization, the patient missed the second treatment at day 7 (V3), but returned to complete the final visit at day 10 (V4). Two patients of the reference group were excluded from PP analysis after day 1 (V2) because they used additional head louse treatments.

Baseline characteristics are presented in Table 1. Most participants were children and female. Adults (18 years and older) accounted for about 20% of participants. In general, both treatment groups were comparable. The proportion of children aged 2 to 6 years was higher in the test group, as compared to the control group. In total, 33 patients had thin hair, distributed similarly to the treatment groups (Table 1).

**Table 1:** Baseline demographic and clinical characteristics for both study groups

		Dimeticone group (n = 50)	Permethrin group (*n* = 50)
Age (years)	Mean (SD)	14.08 (13.7)	15.2 (11.7)
	Median (Q1-Q3)	9.5 (6.0–13.7)	10.5 (8.1–13.7)
Age (groups)	2 to < 6 years (n, %)	13 (26.0)	3 (6.0)
	6 to < 11 years (n, (%)	19 (38.0)	24 (48.0)
	11 to < 18 years (n, %)	8 (16.0)	12 (24.0)
	≥ 18 years (n, %)	10 (20.0)	11 (22.0)
Sex	Male (n, %)	6 (12.0)	8 (16.0)
	Female (n, %)	44 (88.0)	42 (84.0)
Childbearing Potential^a^	Yes (n, %)	21 (47.7)	24 (57.1)
	No (n, %)	23 (52.3)	18 (42.9)
Ethnicity/skin color	Caucasian (n, %)	47 (94.0)	49 (98.0)
	Other (n, %)	3 (6.0)	1 (2.0)
Hair length	Short (n, %)	8 (16.0)	8 (16.0)
	Shoulder long (n, %)	24 (48.0)	22 (44.0)
	Mid back (n, %)	18 (36.0)	20 (40.0)
	Long (n, %)	0 (0)	0 (0.0)
Hair color	Black (n, %)	5 (10.0)	2 (4.0)
	Blonde (n, %)	19 (38.0)	20 (40.0)
	Brown (n, %)	24 (48.0)	22 (44.0)
	Red (n, %)	1 (2.0)	5 (10.0)
	Grey (n, %)	1 (2.0)	1 (2.0)
Hair thickness	Thin (n, %)	18 (36.0)	15 (30.0)
	Normal (n, %)	22 (44.0)	25 (50.0)
	Thick (n, %)	10 (20.0)	10 (20.0)
Hair type	Straight (n, %)	33 (66.0)	30 (60.0)
	Wavy (n, %)	17 (34.0)	15 (30.0)
	Curly (n, %)	0 (0.0)	5 (10.0)
Severity ^b^	Mild 5–9 lice (n, %)	26 (52.0)	20 (40.0)
	Moderate 10–24 lice (n, %)	23 (46.0)	28 (56.0)
	Severe > 24 lice (n, %)	1 (2.0)	2 (4.0)

^a^ Women only

^b^ including adult lice and nymphs

The majority of participants were initially assessed to have a mild or moderate grade of head lice infestation. In general, infestation grades were comparable for both treatment arms. At baseline, patients had a mean number of 1.7 adult lice (SD ± 2.0; range: 0–13) and 9.34 nymphs of all grades. Mean, median and maximum numbers of adult lice and nymphal stages at baseline are depicted by group in Table 2. Maximum number of adult lice and nymphs per patient was 49 in the test group and 41 in the reference group.

**Table 2:** Grade of infestation at baseline (V1)

	Dimeticone group Mean (median; maximum; Q1-Q3)	Permethrin group Mean (median; maximum; Q1-Q3)
Adult lice	1.6 (1.0; 13; 0–2)	1.9 (2.0; 7; 1–3)
Nymphal instars 1	3.6 (3.0; 21; 2–5)	4.0 (3.0; 13; 2–4)
Nymphal instars 2	3.6 (3.0; 15; 2–5)	3.6 (3.0; 14; 2–4)
Nymphal instars 3	1.7 (2.0; 8; 0–3)	2.2 (2.0; 7; 1–3)

### Primary outcome

In the FAS population, all participants in both groups were cured from head lice infestations on day 10 (100% cure rate, Table 3). In the reference group, one participant was assessed to have a re-infestation. Both treatment products achieved the primary objective of superiority to the pre-defined cure rate of > 70% (*p*-value < 0.001).

**Table 3:** Cure rates at day 1, day 7, and day 10 (FAS analysis)

	Dimeticone group	Permethrin group
Day 1 (V2)	49/50 (98.0%)	42/50 (84.0%)
Day 7 (V3)	40/48 (83.3%)	35/48 (72.9%)
Day 10 (V4)	49/49 (100%)	48/48 (100%)

Non-inferiority of the test product could be concluded for both PP (Miettinen-Nurminen Score: 95% CI = [− 7.41; 7.41]; *p* = 0.025) and FAS populations (Miettinen-Nurminen Score: 95% CI = [− 7.27; 7.41]; *p* = 0.024). These confidence limits are greater than the pre-defined margin of − 7.5, and therefore, the null hypothesis was rejected, and non-inferiority was concluded.

At day 1 (V2) efficacy of the test product (98.0%) was significantly higher (95% CI = 89.35–99.95%), as compared to the control group (84.0%; 95% CI = 74.75–95.27%; *p* = 0.031). For mild and moderate infestation rates, the test product showed a higher efficacy, as compared to the control group (98% versus 87.5%), albeit not statistically significant (*p* = 0.059).

In the dimeticone group, within a post hoc analysis, one adult louse and 1 second instar (N2) nymph were found at day 7, but no live lice or nymphs at day 1 (V2). However, the N2 nymph detected most likely resulted from eggs that survived treatment and therefore a re-infestation is not assumed.

Hair characteristics of participants, such as hair length, hair color, or hair type had no effect on the efficacy of the products tested when baseline infestations were of mild or moderate severity (for all parameters *p* > 0.5; Kruskal-Wallis-Test). Due to the low number of severe baseline infestations, the possible effect of hair characteristics on efficacy could not be evaluated.

We found that 39 patients of the 100 patients included in the trial reported at least at one of the visits no additional combing. A total of five patients reported no combing at all throughout the course of the study. All these five patients were cured from head lice. Evaluation for independence of the variables for each of the visits revealed no statistically significant association between cure rates at V2 (*p* = 0.67) and V3 (*p* = 0.69), and the implementation of combing by the participants or their guardians. Twenty seven patients reported no combing at V4, but the effect could not be evaluated statistically, as no treatment failures occurred.

### Adverse events

After adjustment for pre-treatment signs and symptoms (PTSS), a total of 26 adverse events (AEs) occurred in 11 patients in the dimeticone group, and 16 AEs in 12 patients in the permethrin group. One patient treated with the test product presented a serious AE (atypical pneumonia), which was also initially considered a serious adverse device effect which led to the drop-out of the patient for V3. A detailed evaluation revealed that the SAE was not product-related.

Most AEs classified as related to treatment included skin and subcutaneous disorders. Erythema occurred in 3 participants of the test and 7 of the reference group. Pruritus was reported by 4 participants of the test group and 3 of the reference group. Skin burning was stated by 2 participants treated with the test product, and 3 treated with the reference product. Six patients of the test group and one patient of the reference group reported paresthesia after treatment.

The AEs were mild in 30 of 42 documented cases; this refers to 20 AEs in the test group and to 10 AEs in the reference group. Moderate severity was noted for 10 AEs (test group 4 AEs; reference [6] AEs). All other events were classified as unrelated or unlikely to be related with the products.

Two patients received specific medication for treatment of AEs (atypical pneumonia in the test group; conjunctivitis in the reference group). All other AEs resolved spontaneously without any specific treatment.

### Secondary outcomes

#### Global tolerability

Global tolerability was mostly assessed as “very good” by participants or their guardians, followed by “good” in both treatment arms (Table 4).

**Table 4:** Global tolerability assessed by patients/their guardians and blinded investigator via 4-point VRS

	Patient		Investigator	
	Dimeticone	Permethrin	Dimeticone	Permethrin
Very good	41/49 (85.4%)	41/48 (85.4%)	48/49 (98.0%)	44/48 (91.7%)
Good	6/49 (12.2%)	6/48 (12.5%)	–	4/48 (8.3%)
Moderate	2/49 (4.1%)	1/48 (2.1%)	1/49 (2.0%)	–
Poor	–	–	–	–

Only a few patients (3.1%) stated “moderate” global tolerability. Global tolerability as assessed by the investigators was “very good” in nearly all patients, only in a few patients (4.0%) “good” or “moderate” (2.0%) global tolerability was documented. In total, both treatments showed a comparable global tolerability, which was “very good” or “good”, none of the products was assessed to be of poor tolerability.

#### Local tolerability

Pruritus, burning sensation and paresthesia after treatment are presented in Table 5. Burning sensation and paresthesia occurred in a comparable number in both treatment groups. Pruritus was more common in the reference group, which is reflected by the more pronounced improvement of pruritus at visit 1 after treatment in the test group (73.5% improvement), as compared to the reference group (62.0% improvement).

**Table 5:** Local tolerability assessed after treatment in both study groups at any visit (total events)

	Dimeticone group	Permethrin group
Pruritus		
Mild	37	52
Moderate	4	6
Severe	0	1
Burning		
Mild	3	2
Moderate	0	2
Severe	0	0
Paresthesia		
Mild	12	7
Moderate	0	0
Severe	0	0

#### Skin irritation

There was a good skin tolerability in both groups. Erythema occurred more often in the reference group (12% versus 16% after first treatment and 2.1% versus 8.3% after second treatment). No secondary infections were reported at any point in time during the trial.

#### Esthetical effects

The majority of patients/their guardians was satisfied with the esthetical effects of the treatments. In the test group, 90% of patients agreed with the statement “hair looks good”, as compared to 96% in the reference group. The item “hair feels well-groomed” was agreed less commonly by patients that received the test product (88% versus 96%). The item “scalp feels pleasant” was slightly more agreed by patients treated with the test product than by patients treated with the reference product (66.7% versus 64.6%).

## Discussion

Our in vitro bioassays and the clinical trial have shown that the dimeticone-based pediculicide is a safe and efficacious treatment against head louse infestations. After brief exposure time, the product produced a 100% cure rate. This result is particularly important in the current situation of increasing head lice resistance to the permethrin-based OTC formulations available. We consider our study population in the randomized trial as representative of the general population with head louse infestations in Germany that seek care at clinical centers, because the only exclusion criterion that led to non-inclusion was the absence of active infestation.

Technological constraints in the treatment of head lice, such as product concerns, treatment techniques, and resistance to products, are important head lice management issues, as perceived by parents [39]. An excellent safety profile and short application time may increase patients’ and parents’ compliance. In this context, the dimeticone product fills a critical gap: it is a rapid, highly efficacious and safe treatment, based on comprehensive evidence. Data available include description of the precise mode of action, appropriately designed clinical trials, pediculicidal and ovicidal bioassay data, and evidence a high efficacy even in the case of high parasite load and in the presence of resistant strains [23, 31, 40, 41].

The in vitro bioassays have shown 100% pediculicidal activity after an application time of only 10 min. These findings corroborate previous in vitro studies with a dimeticone-based product and an application time of 20 min [31] and support data showing that the product kills head lice within minutes. The criteria for determination of mortality followed standard protocols of the laboratory. Even after applying more stringent criteria for the survival of lice, the results were similar.

An explanation of the extremely rapid onset of the lethal effect has been provided previously by Richling and Böckeler (2007) [23]: the high concentration dimeticone product penetrates into the respiratory system of head lice in less than 1 minute and vital signs disappear after dimeticone completely fills the oxygen-supplying tracheae of the louse’s head. Similarly, ovicidal activity was almost 100%, following the brief exposure time of 10 min. Increasing the exposure time to 20 or 30 min only marginally increased the efficacy (data not shown). Simulation of practical application by testing the activity using a spray protocol instead of immersion of eggs did not substantially change the ovicidal activity of the test product. These results confirm data from previous in vitro bioassays in which exposure times slightly longer than 10 min were used [31]. The consistently high ovicidal efficacy of the dimeticone test product can be explained by the extremely low viscosity of one dimeticone acting as a vehicle for the other dimeticone with a slightly higher viscosity, facilitating the rapid entry of both into the aeropyles of eggs, similar to the entry into the spiracles of lice. The current findings of our randomized trial validate the in vitro data.

In comparison to other pediculicides with a physical mode of action, the test product had a substantially improved efficacy only after one treatment, with 98% of patients assessed to be free from lice 24 h post treatment. In previous studies using comparable study protocols, a mineral oil-based pediculicide had a cure rate of 90% after one treatment [24], and a 4% dimeticone-based product was assessed to achieve a cure rate of 69.8% after a single 15 min application [42].

Interestingly the permethrin-based reference product also showed high efficacy in our trial, where 84% of patients were assessed to be free from lice at day 1, following a single treatment. This observed efficacy for the reference product was higher than expected. Geographically distinct patterns of permethrin resistance are not fully understood so far, but obviously, local German lice populations do not display high levels of resistance, as observed for head lice in other countries such as the U.S. Treatment of head louse infestations with products based on a physical mode of action, like dimeticones, which is the treatment of choice in Germany, has likely decreased the selection pressure on head louse populations from the permethrin-based pediculicides, thereby reducing the level of resistance in local populations. Also, the permethrin-based product used in Germany is not the same as the permethrin-based product used in the U.S.

The majority of patients (97.0%) was assessed to have mild to moderate grades of infestation at baseline, which is in line with current literature and clinical trials performed in Europe. Sex distribution and grade of infestation at baseline were comparable in both treatment groups. After random allocation to one of the two treatment groups, the proportion of children in the age group 2 to 6 years was higher in the test product group when compared to the reference product. Evaluation of the hair structure of the patients, however, revealed that there was no relevant difference in the allocation of patients with the hair characteristic “thin” to either of the treatment groups. Furthermore, all hair characteristics documented had no influence on the efficacy of the products tested.

As one key element of successful eradication of head louse infestations is the correct administration of the product, treatment at the study center by well-trained personnel diminished the risk of application errors, as compared to administration at home by the parents. The strict adherence to the instructions for use might, in part, explain the unexpected high cure rates observed for the permethrin reference product. Furthermore, application time for the reference product within this study was 45 min, which is at the top end of the recommended application time range of 30 to 45 min. The fact that efficacy in the permethrin group was better than expected from reports on similar preparations might be attributed, in part, to absence of local resistance.

At day 7 (V3), treatment with the test product resulted in a cure rate of 83.3%. Nymphs hatching from treated and apparently dead eggs may not be able to develop to adults. Even if they become fully matured, they may not be fertile or display reduced longevity, as shown in former studies. Strycharz et al. (2002) showed that nymphs hatching from dimeticone-treated eggs showed reduced longevity. After 10 min exposure of eggs to a dimeticone-based product, 16% of eggs hatched, but from these only 7% of nymphs reached adulthood and, furthermore, were observed to have a reduced longevity in comparison to controls. Thus, apoptotic processes may have been initiated early on in the louse’s development because of the stress incurred during embryonic development after treatment with a dimeticone-based product. Consequently, reduced adult longevity would result in the production of substantially fewer eggs during the shorter adult lifespan [31].

Pruritus showed a more pronounced decrease after treatment within the group of patients receiving dimeticone, possibly reflecting the higher cure rates observed for the test product at day 7. Both the test and the reference products were assessed to be safe and well-tolerated, with the majority of AEs being classified as mild. One patient treated with the test product presented a serious AE (atypical pneumonia). Although initially evaluated as “possibly related” by the principal investigator, a causality between the SAE and the treatment with the test product was later considered as unlikely, as the SAE occurred 5–6 days after treatment, and independent from an initial mild erythema and allergic reaction that resolved without medical treatment shortly after occurrence.

In general, there are several safety concerns of the group of pediculicides with a neurotoxic mode of action, such as transcutaneous resorption of the active ingredient [43], development of hypersensitivity against pyrethroids, severe neurological complications after accidental ingestion, and increased risk for the development of childhood leukemia [44]. According to the Co-ordination Group for Mutual Recognition and Decentralised Procedures – Human (CMDh), warnings had to be implemented in the respective summary of permethrin products characteristics in order to maintain a positive risk-benefit profile [45]. The information on the risk for hypersensitive reactions had to be included and for the risk of systemic intoxication in children 2–23 months of age the need for a close medical supervision had to be stated. In this context, dimeticone-based products have the advantage that they do not bear these risks and that they are effective, also in the presence of pyrethroid resistance.

### Study limitations

In general, in vitro test conditions only partially reflect in vivo conditions. To better mimic the in vivo situation, for ovicidal testing we applied the products using a spray method, in addition to the traditional immersion procedure. However, the differences between the methods, did not influence the high ovicidal efficacy observed throughout the experiments.

For technical reasons, eggs in different developmental stages were used in the experiments on ovicidal activity. It would have been preferable to perform the tests with batches of eggs with defined developmental characteristics, such as developed eye spots and embryonic movements. A previous study showed that the ovicidal activity of dimeticone was not influenced by the development stage of the embryo. However, with other dimeticone products and treatment options based on a neurotoxic mode of action hatchability depended on developmental stages [41].

The use of a nit comb within clinical trials testing products to control head louse infestation is controversial, as efficacy of the products tested may be affected. This is definitely the case for therapeutic wet combing, but is less of concern with diagnostic dry combing. In a post hoc analysis, we did not find any statistically significant association between the cure rates and the implementation of combing, and all patients that did not use a nit comb throughout the course of the study were cured.

To have treatment protocols as similar as possible for both groups of patients, the restriction not to wash the hair within 3 days after application of the treatment was followed for all patients, even though this restriction resulted from the instruction for use only for the reference product and was to the disadvantage of the test product. The minor differences observed in the evaluation of the esthetical effects may have therefore resulted from dimeticone residues in the patient’s hair, which would have been washed off, if the test product instructions had been followed.

## Conclusions

We have shown a high level of safety and efficacy following brief application of a new dimeticone-based product, as evidenced by in vitro bioassays and a randomized controlled trial. The short application time and the high efficacy, also in the case of resistance, are key drivers for improved patients’ compliance.

## Data Availability

The final clinical study report (CSR) contains full names and addresses of the study personnel involved, as well as commercially confident information. Therefore, a blackened version of the CSR will be made available only upon reasonable request (Dr. Kristina Röschmann, Director Clinical Research, G. Pohl-Boskamp GmbH & Co. KG Germany, k.roeschmann@pohl-boskamp.de).

## Abbreviations

AE: Adverse Event
BfArM: German federal Institute for Drugs and medical Devices
CRO: Contract Research Organization
eCRF: Electronic case Report Form
FAS: Full Analysis Set
GCP: Good Clinical Practice
ICF: Informed Consent Form
ICH: International Conference on Harmonization
IfSG: German Infection Protection Act
Kdr: Knockdown Resistance
min: Minute
ml: Milliliter
mm: Millimeter
PP: Per Protocol
PTSS: Pre Treatment Signs and Symptoms
RCT: Randomized Controlled Clinical Trial
SAE: Serious Adverse Event
SAP: Statistical and Analytical Plan
SD: Standard Deviation
U.S.: United States of America
V: Visit
VRS: Verbal Rating Scale
